# Quasi-homogenous photocatalysis of quantum-sized Fe-doped TiO_2_ in optically transparent aqueous dispersions

**DOI:** 10.1038/s41598-021-96911-6

**Published:** 2021-09-03

**Authors:** Marcus Einert, Pascal Hartmann, Bernd Smarsly, Torsten Brezesinski

**Affiliations:** 1 Surface Science Laboratory, Department of Materials and Earth Sciences, Technical University of Darmstadt, Otto-Berndt-Str. 3, 64287 Darmstadt, Germany; 2 Institute of Physical Chemistry, Justus-Liebig-University Giessen, Heinrich-Buff Ring 17, 35392 Giessen, Germany; 3 Institute of Nanotechnology, Karlsruhe Institute of Technology (KIT), Hermann-von-Helmholtz-Platz 1, 76344 Eggenstein-Leopoldshafen, Germany

**Keywords:** Nanoparticles, Photocatalysis, Electrochemistry

## Abstract

In this study, the preparation of anatase TiO_2_ nanocrystals via a facile non-aqueous sol–gel route and their characterization are reported. The 3–4 nm particles are readily dispersable in aqueous media and show excellent photoreactivity in terms of rhodamine B degradation. The catalytic performance can be further increased considerably by doping with iron and UV-light irradiation as a pre-treatment. The effect of surface ligands (blocked adsorption sites, surface defects etc.) on the photoreactivity was thoroughly probed using thermogravimetric analysis combined with mass spectrometry. Photoelectrochemical characterization of thin-film electrodes made from the same TiO_2_ nanocrystals showed the opposite trend to the catalytic experiments, that is, a strong decrease in photocurrent and quantum efficiency upon doping due to introduction of shallow defect states.

## Introduction

The wide-bandgap semiconductor titanium dioxide, TiO_2_, is the most widely investigated photocatalyst for the decomposition of organic contaminants and water splitting^1,2^. At the same time, TiO_2_ is one of the most promising transition-metal oxides for industrial catalytic applications due to its unique optical and electronic properties, high chemical stability, negligible toxicity and relatively low costs^3,4^. Overall, TiO_2_ is still heavily researched by materials scientists across the world and is often utilized as a benchmark or reference material^5^. The photooxidation of organic species by TiO_2_ would be beneficial for industrial purposes, such as the control of ground water contamination^6,7^ or reduction of air pollution^8^. The major advantage of using TiO_2_ in water purification compared to conventional technologies, such as activated carbon adsorption, is the complete mineralization to CO_2_ and H_2_O combined with low processing costs^9,10^. However, the degradation rate of many organic pollutants (e.g., dioxins) when using common photocatalysts is too low for practical applications^11^. For increasing the activity of catalysts in heterogeneous photocatalysis, a high dispersability in the reaction medium is desirable to avoid light scattering processes at the surface by suspended particles^12,13^. Note that the activation of bulk particles requires an isotropic medium that provides a sufficient light penetration depth^14,15^.

The relatively large bandgap of anatase TiO_2_ (*E*_gap_ ≈ 3.2 eV), corresponding to wavelengths shorter than ~ 390 nm, allows < 4% of solar energy to be converted into electrical energy. The most effective way of achieving an absorption red shift in the photocatalyst, thereby enhancing the generation of photoexcited charge carriers, has been found in the doping of TiO_2_ with various cations^16^. Metal-ion dopants substitute the Ti^4+^ site in the crystal lattice, creating discrete energy levels within the electronic band structure^17^. Iron has been demonstrated to be one of the most efficient dopants for increasing the photoreactivity^18^. Iron-doping induces energy levels between 0.2 and 0.4 eV above the valence-band edge, as shown for quantum-sized TiO_2_ particles^19^. Furthermore, large improvements in photoreactivity have been observed through nanostructuring^20–22^ and substitutional/interstitial anion doping with nitrogen^23^ and sulfur^24,25^, shifting the optical absorption spectrum into the visible range^26^.

Since Niederberger et al. applied the general concept of non-aqueous sol–gel chemistry to the preparation of TiO_2_ nanoparticles in 2002^27^, several authors reported about modified low-temperature synthesis routes of anatase TiO_2_ for photocatalytic applications^28–33^. For example, Zhang et al. reported about improved photoreactivity of 15 nm TiO_2_ particles and proved their long-term stability as a dispersion in water^32^. However, to the best of our knowledge, there are no studies available on the preparation and photocatalytic performance of (non-aqueous) sol–gel derived Fe-doped TiO_2_ nanocrystals having a size below 5 nm. It is worth mentioning that apart from photocatalysis, the surface composition of such particles can be tailored toward the fabrication of well-defined mesoporous^34–36^ or macroporous thin-film electrodes^37^ for electrochemical applications^38,39^.

In this study, the photocatalytic activity of undoped and Fe^3+^-doped TiO_2_ nanocrystals synthesized via a facile benzyl alcohol-based sol–gel route was examined regarding the bleaching of rhodamine B (RhB). The 3–4 nm particles exhibited a high dispersability in aqueous media (up to 10 wt-%), forming colloidal dispersions that were stable over days. In addition to nanostructuring and doping, emphasis is placed on the correlation between ultraviolet (UV)-light irradiation as a kind of pre-activation and photoreactivity. It is demonstrated that the TiO_2_ nanocrystals show significantly improved photodegradation kinetics after irradiation due to the removal/stripping of organic surface ligands.

## Results

Considering the parameters discussed in the Introduction section, photocatalyst materials need to be tailored in terms of particle size, bulk and surface composition, degree of crystallinity and dispersability. The photocatalysts employed in this work were prepared by a non-hydrolytic benzyl alcohol-based synthesis route^40–43^, allowing the formation of both undoped and doped TiO_2_ nanocrystals, the latter by incorporation of Fe^3+^ ions during the condensation reactions. In recent years, Fe(NO_3_)_3_·9H_2_O for cation doping has been shown to be a suitable precursor due to the relatively weak adsorption of NO_3_^−^ on the particle surface^44^. To study the effect that the iron doping level has on the photoreactivity, TiO_2_ nanocrystals of different composition were prepared and tested. The amount of incorporated dopants was determined by energy-dispersive X-ray spectroscopy (EDS) and found to be ~ 1.0, 1.5 or 4.3 mol.% (5% error). Overall, EDS indicated the presence of Ti, O, Cl and Fe (see Supplementary Figure S1). In this context, we also note that the actual Fe^3+^ doping level deviated strongly from the amount of precursor used in the synthesis (e.g., 1.5 versus 7.5 mol.%).

**Figure 1:**
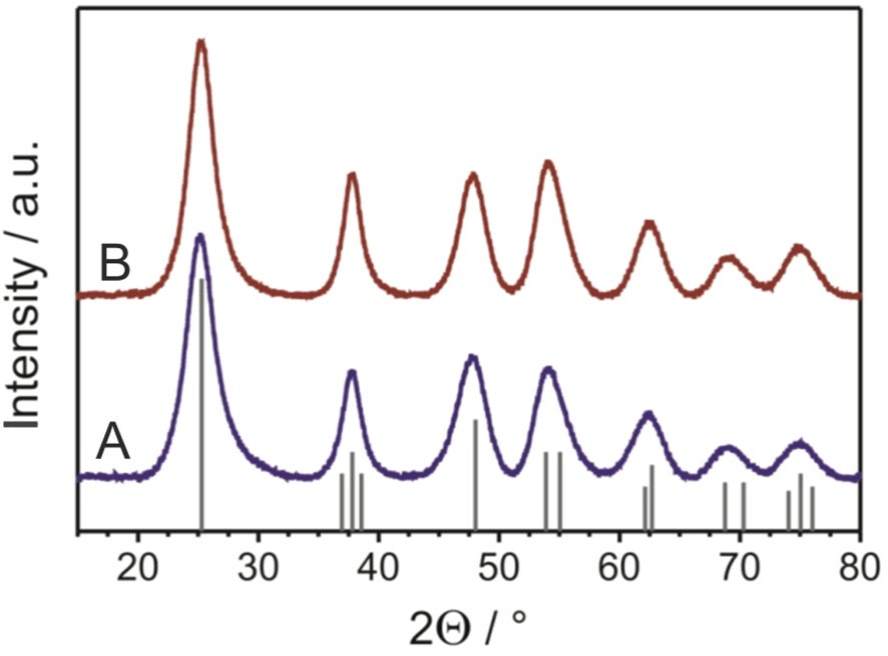
XRD patterns of the undoped **(A)** and 1.5 mol.% Fe^3+^-doped **(B)** TiO_2_ nanocrystals. The line pattern shows JCPDS reference card no. 21-1272 for anatase TiO_2_.

X-ray diffraction (XRD) patterns of the undoped and Fe^3+^-doped TiO_2_ nanocrystals (see Fig. 1 and Supplementary Figure S2) were found to match the anatase phase of TiO_2_ (JCPDS card no. 21–1272). The pronounced reflections confirm the crystallinity of the TiO_2_ samples. This result is noteworthy, as sol–gel derived materials often require some kind of post-treatment for achieving a reasonable degree of crystallinity. Furthermore, no α-Fe_2_O_3_ (note that hematite is the thermodynamically most stable iron-oxide phase)^45^ reflections were observed, supporting the assumption that Fe^3+^ (0.64 Å, octahedral coordination, high-spin state) substitutes the Ti^4+^ (0.605 Å) site in the lattice^46^. The average crystallite size was determined by applying the Scherrer equation to the full width at half-maximum (FWHM) intensity of the 101 reflection at 2θ = 25.2°. It was found to be 3.5 nm for both the undoped and Fe^3+^-doped TiO_2_ materials (i.e., the doping level has no notable effect on the crystallite size). For such small-size particles, high photocatalytic activities have been observed, as the electrons and holes after optical excitation can readily react with the adsorbate on the surface of the adsorbent if the dopant acts as a mediator of interfacial charge transfer^47^. Taken together, XRD provides clear evidence of the crystalline nature of the TiO_2_ nanoparticles, which is required for proper charge-carrier migration to the surface (interface). Amorphous photocatalysts usually show lower photocatalytic activity due to the presence of abundant defect sites, acting as recombination centers^48^.

To probe the dispersability of the TiO_2_ nanocrystals in water, dynamic light scattering (DLS) measurements were performed (see Supplementary Figure S3). DLS indicates a narrow particle size distribution with an average hydrodynamic diameter of (12.5 ± 2.6) nm. This result suggests that the nanoparticles tend to form agglomerates in polar solvents, which is reasonable from a surface-energy perspective and is in agreement with electron microscopy imaging data (see Supplementary Figure S4). In addition, selected-area electron diffraction (SEAD) confirmed the phase purity of the anatase TiO_2_ nanoparticles.

Because the specific surface area plays an important role in catalytic applications, N_2_-physisorption measurements were conducted on the TiO_2_ nanocrystals. The adsorption/desorption isotherms (see Supplementary Figure S5) show a combination of H2- and H4-type hysteresis^49^. In general, H2 hysteresis is attributed to condensation of adsorbate in porous materials that show pore blocking/percolation effects. In contrast, H4 hysteresis can be primarily assigned to cavitation-induced evaporation (in mesoporous and microporous materials)^50^. Both can be somewhat expected, as the aggregation of TiO_2_ nanocrystals inevitably leads to interparticle void formation. Brunauer–Emmett–Teller (BET) analysis provided a specific surface area of *A*_BET_ ≈ 260 m^2^/g for both the undoped and Fe^3+^-doped TiO_2_ nanocrystals. This result agrees well with theoretical calculations assuming a cubic close-packed arrangement of 3.5 nm spherical particles.

**Figure 2:**
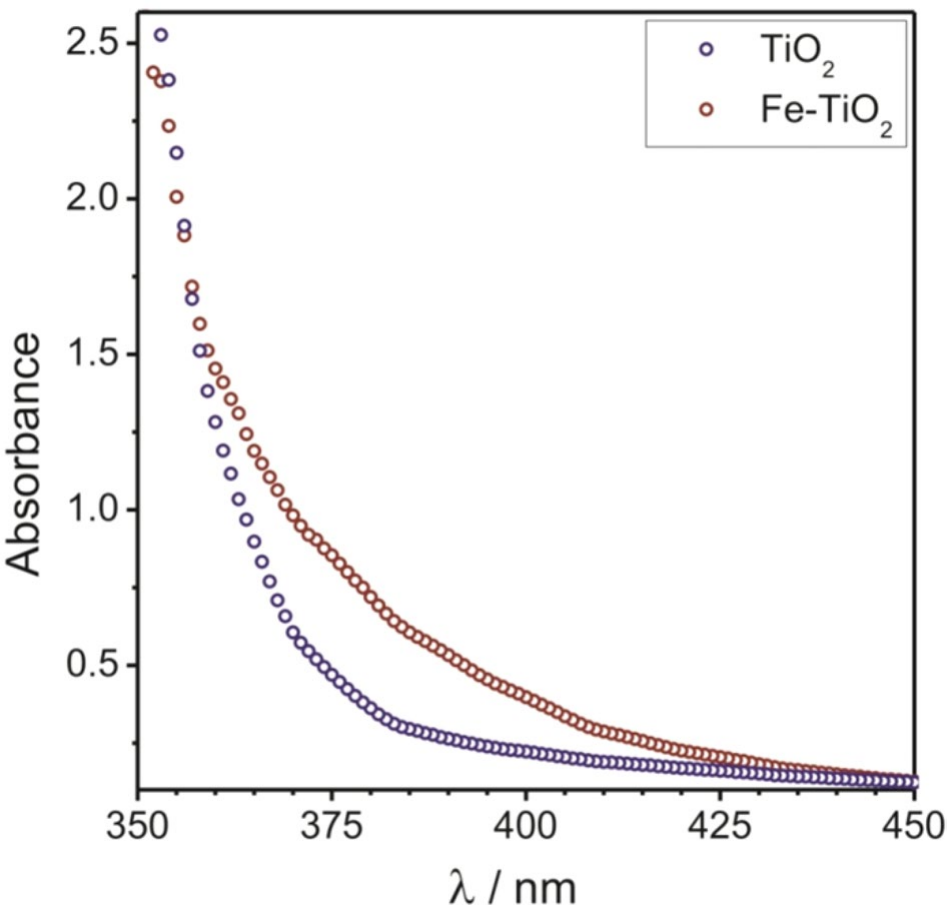
Optical absorption spectra for the undoped (purple) and 1.5 mol.% Fe^3+^-doped (red) TiO_2_ nanocrystals.

Figure 2 shows optical absorption spectra for the undoped and Fe^3+^-doped TiO_2_ nanocrystals. The data indicate that the doped material absorbs light much stronger at wavelengths shorter than 400 nm than the undoped counterpart does. The 1.5 mol.% Fe^3+^-doped TiO_2_ nanocrystals exhibited the largest optical absorption properties (see Supplementary Figure S6). The intrinsic band-edge absorption due to interband transition from the O-2p to Ti-3d orbitals of undoped titania^51^ can be found in the range between 375 and 340 nm. The stronger absorption of the doped material (red shift) between 450 and 355 nm results from the excitation of electrons from the Fe-3d states to the TiO_2_ conduction band^52^. For TiO_2_ as an indirect bandgap semiconductor, the square root of the absorption coefficient is expected to vary linearly with photon energy near the bandgap energy. The optical bandgaps were determined from the respective Tauc plots (see Supplementary Figure S6) to be about 3.4 eV (365 nm) and 3.3 eV (376 nm) for the undoped and Fe^3+^-doped TiO_2_, respectively. The fact that *E*_gap_ is larger by 0.1–0.2 eV compared to that of bulk anatase TiO_2_^51^ can be ascribed to nanoconfinement effects, in agreement with the bandgap shift of 0.16 eV for 3.8 nm TiO_2_ particles observed by Anpo et al.^53^ The theoretical calculation model for bandgap shifts of quantum-sized particles was first described by Brus et al. in 1984^54^. The nanoconfinement effect, in general, can be explained by Heisenberg’s uncertainty principle: As the particle size decreases, the ground state energy of confined electrons must increase to satisfy the uncertainty principle ($\Delta x\cdot \Delta p \le \frac{\hslash }{2}$).

**Figure 3:**
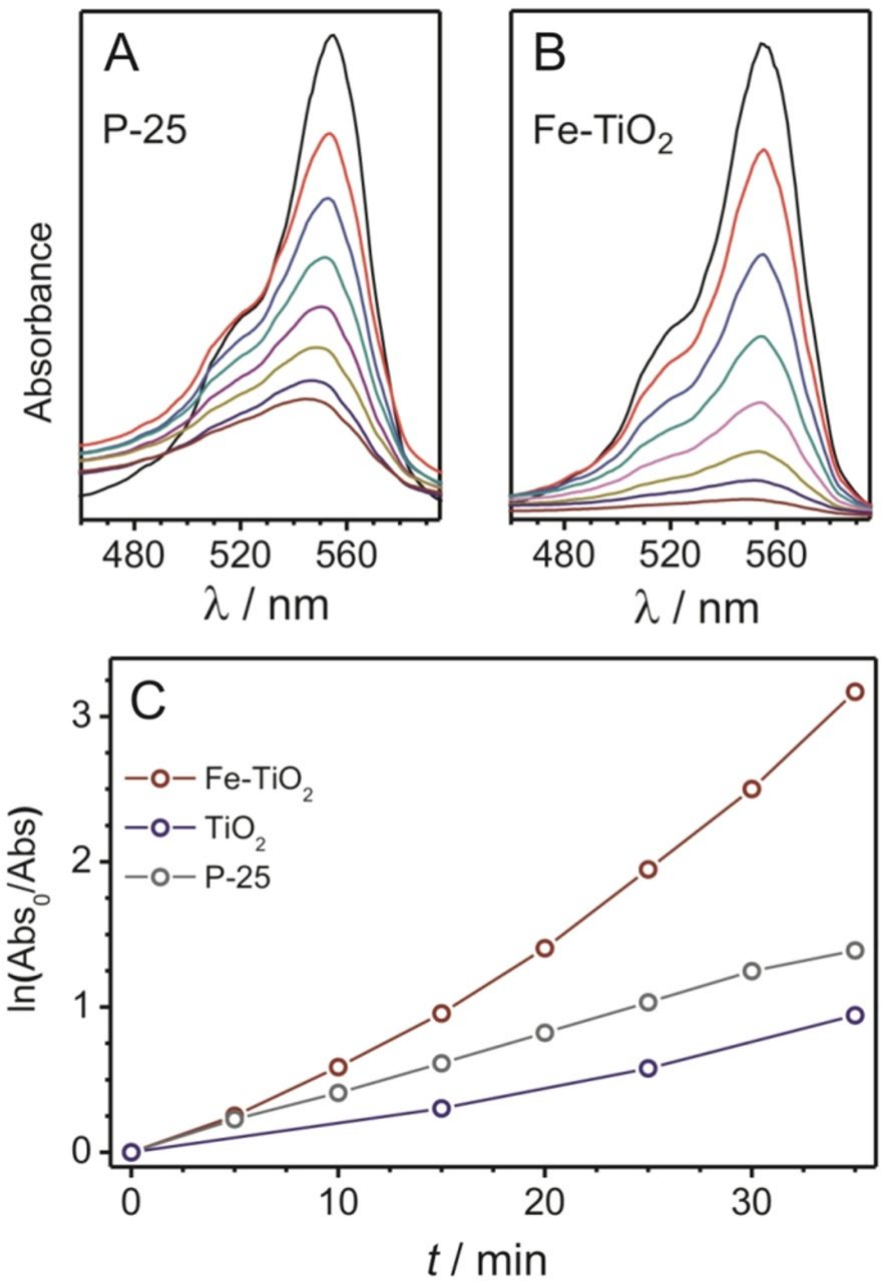
UV–vis spectra showing the photobleaching of 40 mL aqueous RhB solutions (25 μmol/L) in the presence of P-25 as a reference TiO_2_ photocatalyst **(A)** and 1.5 mol.% Fe^3+^-doped TiO_2_ nanocrystals **(B)** upon UV-light irradiation (35 min in total, 5 min steps). Corresponding semilogarithmic plots **(C)**. The performance of the undoped TiO_2_ nanocrystals is also shown. The connecting lines are for eye guidance.

The photocatalytic activity of the undoped, (1.0, 1.5 and 4.3 mol.%) Fe^3+^-doped and anatase/rutile P-25 (Degussa) TiO_2_ was studied by monitoring RhB [as a representative environmental (organic) pollutant] degradation under UV-light irradiation [< 254 nm, see UV–visible (UV–vis) spectra in Fig. 3A,B]. The 1.5 mol.% Fe^3+^-doped TiO_2_ was found to be the most active photocatalyst among the materials tested in this work (see Supplementary Figure S7) and therefore chosen for further structural/optical investigations together with the undoped TiO_2_ sample. The results in Fig. 3C show that the photobleaching in water follows pseudo-first-order reaction kinetics. The degradation rate, *k*, for the 1.5 mol.% Fe^3+^-doped TiO_2_ was about two times larger ($k=9.0\cdot {10}^{-2 }{\mathrm{s}}^{-1}$) compared to that of undoped TiO_2_ ($k=4.3\cdot {10}^{-2 }{\mathrm{s}}^{-1}$) and the P-25 reference material ($k=4.6\cdot {10}^{-2 }{\mathrm{s}}^{-1}$). This result demonstrates that the photocatalytic activity of TiO_2_ nanocrystals can be substantially improved via doping with iron. Interestingly, the experimental data for the as-prepared TiO_2_ nanocrystals did not show the same linear (kinetics) trend observed for P-25. This finding can probably be explained by the presence of organic surface ligands from the synthesis^41,43^. The ligands may act as trap states for charge carriers at the particle surface and can sterically hinder the adsorption of RhB. The first data point (*t* = 0 min, black lines in Fig. 3A,B) was measured in the absence of photocatalyst material. For the P-25/RhB suspension (Fig. 3A), a sudden increase in absorption at *λ* ≤ 500 nm after *t* = 0 min was observed, which is due to scattering of incident light, especially in the visible range, by the TiO_2_ particles.

**Figure 4:**
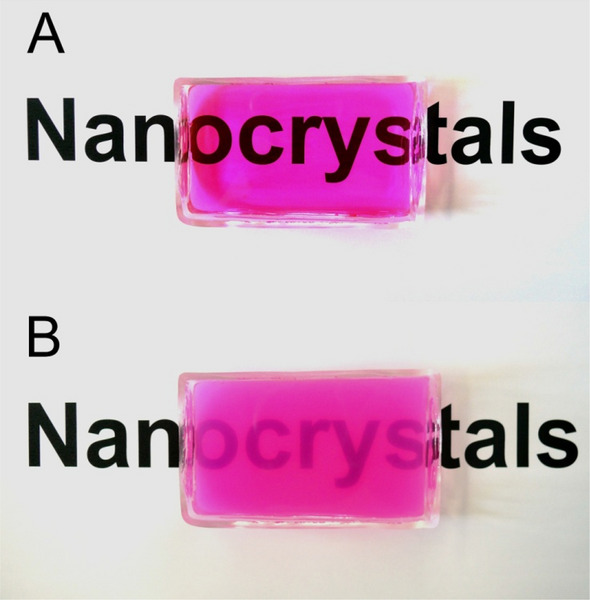
Photographs of 5 mg 1.5 mol.% Fe^3+^-doped TiO_2_ **(A)** and 3.5 mg P-25 TiO_2_ **(B)** dispersed/suspended in 80 mL aqueous RhB solutions (25 μmol/L).

The photographs in Fig. 4 clearly show that the P-25 particles cannot be dispersed well in water, forming a suspension and thereby scattering the incident light to some degree. In contrast, the sol–gel derived TiO_2_ nanocrystals produced an optically transparent (colloidal) solution under the very same conditions. Therefore, it was not necessary to centrifuge the particles for photocatalytic measurements.

The degradation rate has been reported to be proportional to the incident light intensity below 20 mW/cm^2^ and exhibit square root dependence above ~ 25 mW/cm^2^^55,56^. This suggests that the photoreactivity is strongly affected by the penetration depth of UV light. The latter was examined for the TiO_2_ nanocrystals dispersed in 80 mL aqueous RhB solution (25 μmol/L, see Supplementary Figure S8). The absorbance of incident photons by the RhB/TiO_2_ dispersion revealed an exponential decay from 300 to 370 nm, meaning that the absorption of high-energy photons is significantly increased. On the other hand, the physical penetration depth decreased from 24 to 2.5 cm between 370 and 300 nm. Hence, to make use of high-energy photons (in an effective manner) for charge-carrier generation in the TiO_2_ photocatalyst, the penetration depth of light and the reactor dimensions have to be taken into account carefully. The water level in the reactor employed in this work had a height of about 3 cm, thus only light of wavelength above 330 nm was capable of addressing the particles near the bottom of the reactor.

In general, the photocatalytic activity of quantum-sized TiO_2_ depends to a large extent on the dopant nature and its concentration^19^. It is known that in nanostructured TiO_2_, Fe ions as dopant act as trapping sites for the photoexcited electron–hole pairs (i.e., the recombination of immobilized charge carriers—spatially separated from each other—is inhibited)^57^, increasing their mean lifetime from the nanosecond to millisecond range^47^. In bulk anatase TiO_2_, electrons may be trapped in the respective states energetically located within the electronic conduction-band structure and holes in the Fe^3+^ states (located within the TiO_2_ bandgap) after photoexcitation^58^. However, for quantum-sized particles, they are at least several tenths of meV below the conduction band due to nanoconfinement-induced bandgap widening. Therefore, recombination of electron–hole pairs is somewhat inhibited, resulting in extended diffusion lengths. The trap-mediated charge transfer is well known to occur through detrapping mechanisms and tunneling processes^47,59^. In a 4 nm TiO_2_ particle (~ 1000 atoms, ~ 2 nm exciton diameter), the majority of dopant atoms are located close to or at the surface^60^. Consequently, trapped electrons and holes may lead immediately to the formation of highly reactive oxygen species, O_2_^•−^, HOO^•^ and/or ^•^OH^61^. Such mediated charge-transfer processes (besides the superior light absorption in the visible range due to introduction of Fe-3d states, see Fig. 2) help to explain the substantially increased photoreactivity observed for the 1.5 mol.% Fe^3+^-doped TiO_2_ as compared to the undoped nanocrystals. The highest photodegradation rate was demonstrated for a doping level of 1.5 mol.% (see Supplementary Figure S7), in good agreement with the study reported by Ranjit et al.^62^ Note that excessive doping does not improve the photoreactivity (as demonstrated for the 4.3 mol.% Fe^3+^-doped TiO_2_ nanocrystals), as the high-concentration dopant states serve as recombination centers (trapped charge carriers recombine through tunneling processes). The more iron is introduced into the host lattice, the more the average distance between the confined dopant states declines, ultimately leading to an exponential increase in recombination rate^19,47,57,58^. It should also be noted that Crişan et al. probed the chemical configuration of surface atoms in sol–gel derived Fe-doped TiO_2_ nanoparticles by X-ray photoelectron spectroscopy (XPS). They showed that the Ti 2p (Ti^4+^), O 1 s (O^2−^) and Fe 2p (Fe^3+^) core-level peaks do not change in terms of binding energy (oxidation state) for doping levels between 0.5 and 5 wt.%^63^.

**Figure 5:**
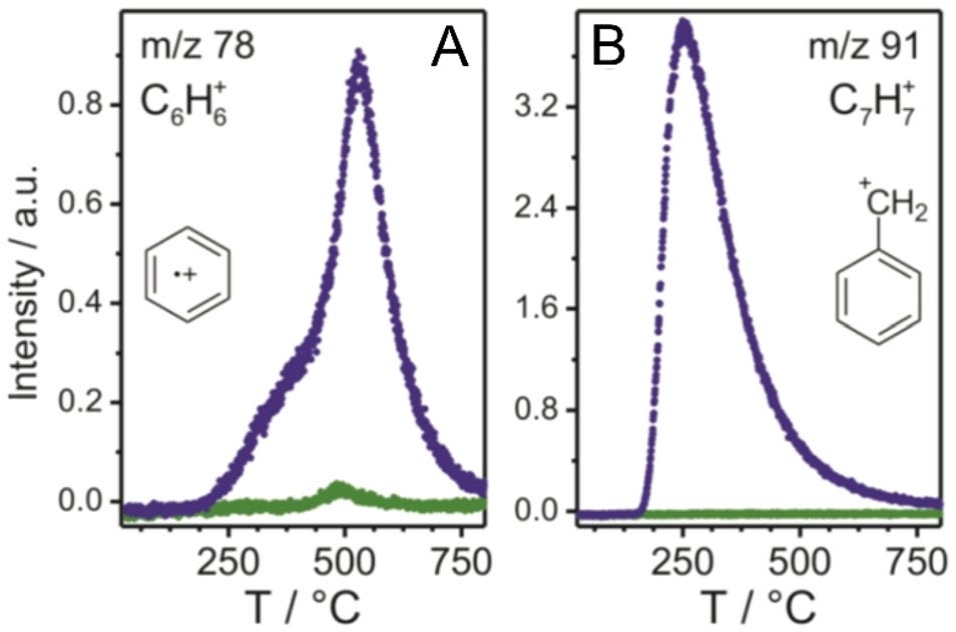
MS signals for the aromatic fragments C_6_H_6_^+^ **(A)** and C_7_H_7_^+^ **(B)** detected in TGA-MS measurements of the as-prepared (purple) and UV-light irradiated (green) TiO_2_ nanocrystals.

Thermogravimetric analysis (TGA) combined with mass spectrometry (MS) was performed to gain insight into the surface composition and to examine to which extent surface-bound ligands affect the photoreactivity of the TiO_2_ nanocrystals. It is know that reaction species, such as benzyl alcohol, diethyl ether, hydroxyl or chlorine ligands, may be present on the TiO_2_ particle surface after synthesis^41^. To determine the amount of organic residues (for vacuum-dried TiO_2_), the weight change during heating was monitored by means of TGA-MS. Supplementary Figure S9 compares the TGA curve of as-prepared material to that of nanoparticles UV-light irradiated for 120 min in deionized water. Interestingly, the mass loss of the irradiated sample was significantly lower (25 versus 30% at 800 °C), suggesting that the surface-bound ligands are decomposed during the treatment (due to the intrinsic photocatalytic activity of TiO_2_^37^). This result is corroborated by the MS data shown in Fig. 5A,B and Supplementary Figure S10. Especially the degradation of benzyl alcohol-type ligands is confirmed by the significantly lower intensity of aromatic fragments, such as C_6_H_6_^+^ (*m*/*z* = 78), C_7_H_7_^+^ (*m*/*z* = 91) and C_5_H_5_^+^ (*m*/*z* = 65), for the UV-light treated material. According to the Spectral Database for Organic Compounds, the tropylium cation is one of the main degradation products of benzyl alcohol. The detection of CO_2_^+^ fragments (*m*/*z* = 44, see Supplementary Figure S10) further supports the presence organic surface contaminants. In addition, the UV-light irradiation removed remaining chlorine ligands (*m*/*z* = 35) from the precursor (see Supplementary Figure S10).

**Figure 6:**
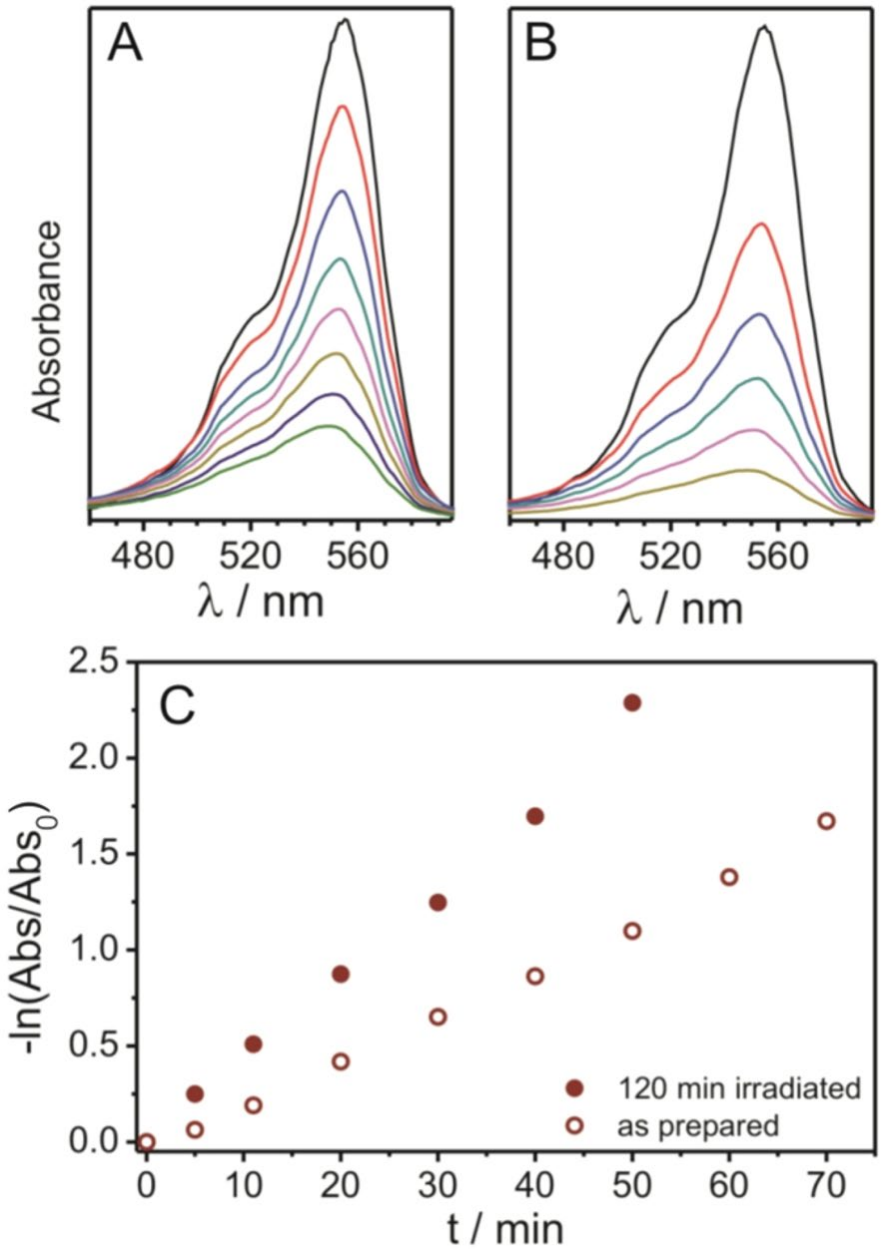
UV–vis spectra showing the photobleaching of 80 mL aqueous RhB solutions (25 μmol/L) in the presence of Fe^3+^-doped TiO_2_ nanocrystals (50/70 min in total, 10 min steps): as-prepared **(A)** and after UV-light irradiation for 120 min in deionized water **(B)**. Corresponding semilogarithmic plots **(C)**.

Figure 6A–C displays the effect that UV-light pre-treatment has on the photoreactivity of the Fe^3+^-doped TiO_2_ nanocrystals. The pre-treated particles showed much improved photocatalytic performance compared to the as-prepared material. We conclude from this data that bulky ligands, such as benzyl alcohol, inhibit the adsorption of RhB and O_2_/H_2_O on the particle surface while acting at the same time as trap states for charge carriers, thereby decreasing the overall photoreactivity. Availability of adsorption sites is a prerequisite for the photocatalytic reactions to occur^1,5^. The radicals formed through redox reactions with O_2_ and H_2_O initiate the photobleaching of RhB by attacking the aromatic chromophore ring structure and inducing de-ethylation and oxidative degradation^64^.

**Figure 7:**
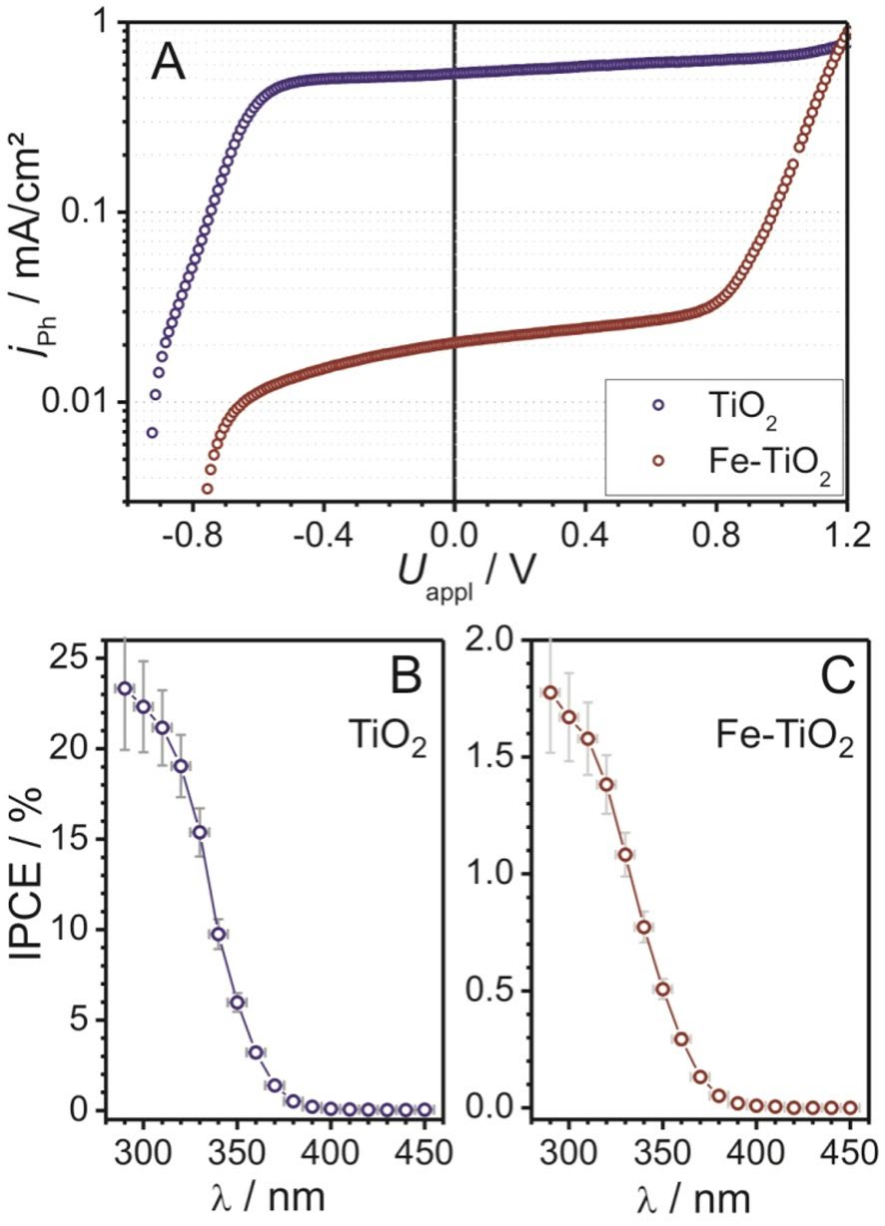
**(A)***I*–*U* characteristics of the undoped and 1.5 mol.% Fe^3+^-doped TiO_2_ nanocrystals deposited onto FTO-coated glass substrates under irradiation (~ 360 mW/cm^2^). Quantum efficiency of undoped **(B)** and doped **(C)** TiO_2_ at *U*_appl_ = 0 V. The connecting lines are for eye guidance.

Apart from photocatalysis, TiO_2_ nanomaterials have been widely investigated in photoelectrochemical applications, e.g., in dye-sensitized solar cells (DSSC)^65^ or for solar hydrogen generation^2^. The latter describes the process of splitting water into hydrogen and oxygen in a simple electrochemical experiment (using a semiconductor electrode and the energy of light irradiation) and was first reported by Fujishima and Honda in 1972^66^. Figure 7A shows the *I*-*U* characteristics of nanocrystal-based TiO_2_ thin-film photoelectrodes on FTO-coated glass substrates. The measurements were carried out at a sweep rate of 2 mV/s. The undoped and 1.5 mol.% Fe^3+^-doped TiO_2_ nanocrystals showed an open cell voltage, *U*_oc_, of about − 0.95 and − 0.8 V (no steady-state value) and a steep increase in photocurrent, *j*_ph_, with increasing applied voltage, *U*_appl_. *j*_ph_ increased only slightly between − 0.5 and + 0.8 V (0.54 and 0.02 mA/cm^2^ short-circuit current densities for the undoped and Fe^3+^-doped TiO_2_, respectively). For *U*_appl_ > 0.8 V, *j*_ph_ strongly increased again. However, this result has no photoelectrochemical origin, as the same behavior was observed without illumination. The maximum conversion efficiency was determined to be 0.18% for the undoped and 0.007% for the Fe^3+^-doped TiO_2_ (at *U*_appl_ = 0 V).

The quantum efficiency or incident photon-to-current efficiency (IPCE) is also commonly used to evaluate the performance of photoelectrodes. Assuming ideal monochromatic light and complete Faradaic conversion, the IPCE can be calculated from the photocurrent response (Fig. 7B,C). As is evident, both samples showed notable IPCEs for wavelengths shorter than 380 nm, with maximum efficiencies of about 23 and 1.8% (within the uncertainty indicated by the error bars) for the undoped and Fe^3+^-doped TiO_2_, respectively. Measurements at lower wavelengths were not feasible for experimental reasons. The large error for small wavelengths is due to the UV fraction in the light-source spectrum (relatively large uncertainty of the intensity measurement).

Although the 1.5 mol.% Fe^3+^-doped TiO_2_ showed superior performance in the photobleaching of RhB, in this kind of photoelectrochemical experiments the doping reduced the efficiency by more than an order of magnitude. In contrast to photocatalysis with nanoparticles (3–4 nm) dispersed in solution, efficient electron transport over extended distances (several hundred nanometres) is required in a photoelectrode*.* As known from literature and as described above, Fe dopants introduce shallow trapping sites, thereby lowering the recombination rates of photoexcited charge carriers. This in turn leads to improved photocatalytic activity as long as the carriers are trapped near the particle surface or diffusion pathways are short. Zhang et al. showed that the optimal iron doping level decreases (from 0.2 to 0.05 at-%) with increasing particles size^67^. In a photoelectrochemical experiment, only efficient “long-distance” charge transport, from the position of the excited site to the electrode back contact, results in high photocurrents and therefore high performance. Egerton et al. studied sol–gel derived TiO_2_ photoelectrodes with varying iron levels and observed a characteristic decrease in photocurrent with increasing content from 0 to 2.2%^68^. This is in agreement with the findings of this work.

## Discussion

In summary, we have applied a non-hydrolytic sol–gel route for the preparation of anatase TiO_2_ nanoparticles that can be readily re-dispersed in aqueous media. The particles had a size of 3–4 nm in diameter, leading to a high specific surface area of ~ 260 m^2^/g. Because of their excellent dispersability, the TiO_2_ nanoparticles were investigated in the photocatalytic degradation of RhB in water. In general, the dispersability provided an ideal experimental setup, with even deeply dispersed particles in the photoreactor being addressable for high overall reactivity. The photocatalytic performance was further improved by a factor of two through appropriate iron doping and pre-irradiation using UV light. TGA-MS measurements showed that the latter leads to surface ligand removal/stripping, increasing the number of active adsorption sites, among others, and therefore the rate of mineralization. However, photoelectrochemical studies revealed that iron doping adversely affects the photoresponse of TiO_2_ thin-film electrodes because of differences in the migration distances of charge carriers (bulk recombination determines the photocurrents of TiO_2_ photoelectrodes).

## Materials/methods

### Materials

Titanium tetrachloride (99.9%), anhydrous benzyl alcohol (99.8%), iron nitrate (FeNO_3_·9H_2_O) and rhodamine B (RhB, dye > 90%) were purchased from Sigma-Aldrich. Ethanol (absolute grade) and methanol were purchased from VWR and propanediol (99%) from Acros Organics.

### Synthesis of TiO_2_ nanocrystals

The preparation of anatase TiO_2_ nanoparticles was adopted from Niederberger et al.^27^ and Brezesinski et al.^34^ 1 mL (9.12 mmol) TiCl_4_ was added dropwise to 5 mL absolute ethanol in a water-free and loosely sealed glass vial. The resulting transparent (pale yellow) solution was combined with 0.25 mL propanediol and mixed with 20 mL benzyl alcohol under vigorous stirring. Finally, the required amount of FeNO_3_·9H_2_O was dissolved into the solution, followed by treatment in an ultrasonication bath to achieve a homogenous and transparent sol. The sol was filtered using a millipore filter (0.2 μm) and then heated at 110 °C for 3 h under constant stirring. To isolate the nanoparticles, the opaque suspension was precipitated in 250 mL diethyl ether, centrifuged at 6000 rpm for 10 min and subsequently dried at ambient conditions. The undoped and Fe-doped TiO_2_ appeared as white and yellow powder, respectively. Transparent aqueous dispersions were obtained by re-dispersion of nanoparticles in deionized water.

### Synthesis of TiO_2_ thin films

Thin films were prepared by the dip-coating method on quartz glass (Lithosil^®^, Schott) or FTO-coated glass substrates. For the dip-coating solution, 500 mg (as-prepared) nanoparticles were dispersed in 4 g methanol and 0.3 g double distilled water. Optimal coating conditions included a relative humidity of 70% and constant withdrawal rates from 3 to 13 mm/s. The as-made films were dried in air at 100 °C for 1 h and then at 300 °C for 12 h. To remove the organic constituents, the samples were heated to 550 °C within 50 min and kept at this temperature for 5 min.

### Characterization

The dopant concentration was probed via energy-dispersive X-ray spectroscopy (EDS, Link Pentafet, 7426, Oxford Instruments) by irradiating a (10 × 10) μm^2^ sample area at 10 kV. X-ray diffraction (XRD) measurements were performed on an X’Pert Pro diffractometer from Panalytical Instruments (Cu Kα radiation) at an acceleration voltage of 40 kV and emission current of 30 mA. XRD data were collected in θ–2θ geometry in the range of 20°–80° in step scan mode (0.008° step size). N_2_-physisorption experiments were carried out at 77 K using a Quantachrome Autosorb instrument. Prior to the measurements, the samples were degassed in a vacuum at 120 °C. For thermogravimetric analysis (TGA), a QMG421 mass spectrometer system (Balzers) combined with a Netzsch STA409PC thermal analyzer were used. Specifically, 10 mg vacuum-dried (24 h) TiO_2_ powder was heated to 800 °C at a rate of 5 °C/min in an oxygen/argon (20:80) atmosphere. The optical properties of nanoparticles were determined by measuring the light absorption spectra of diluted dip-coating solutions (~ 6 mg/mL) using a UVIKON XS spectrophotometer equipped with 3 mL cuvettes.

### Photocatalytic experiments

The photocatalytic activity was measured by monitoring the photobleaching of RhB. In a quartz reactor, 5 mg TiO_2_ nanoparticles were dispersed into 35 or 70 mL deionized water under moderate stirring. Subsequently, 5 or 10 mL aqueous solution of 200 μmol/L RhB was added to the dispersion, yielding a 25 μmol/L RhB solution. The transparent dispersion was aged for 10 min to attain adsorption/desorption equilibrium of dye on the photocatalyst surface, as reported in detail elsewhere^69^. In case of the pre-irradiation treatment, TiO_2_ nanoparticle dispersions were illuminated with a UV lamp (8 W, λ < 254 nm, Benda NU-8 KL) for 120 min to decompose the organic (and inorganic) surface ligands. The distance between the light source and the sample was about 6 cm. The quantitative change in absorbance at 554 nm was measured using an UVIKON XS spectrophotometer.

### Photoelectrochemical experiments

Photoelectrochemical measurements were carried out in a rectangular PMMA reactor [(30 × 50 × 100) mm^3^] equipped with a quartz window. A two-electrode system was used with a Pt foil counter-electrode (300 mm^2^) and the TiO_2_ electrode on FTO-coated glass substrate (19.6 mm^2^) in an aqueous 0.1 M NaOH electrolyte. For bias supply and current measurements, a Zahner IM-6 electrochemical workstation was used. A 150 W Xe Arc lamp (LOT-Oriel) served as the light source. A thermopile and powermeter were used for light-intensity measurements. The incident photon-to-charge carrier conversion efficiency was examined by irradiating the sample with monochromatic light (LOT-Oriel monochromator MSH101, 2.5 mm width of entrance and exit slit). The photocurrent at each wavelength was recorded until steady-state condition was reached (at constant electrode potential of 0 V).

## Supplementary Information

Supplementary Figures.

## Data Availability

The datasets generated during and/or analyzed during the current study are available from the corresponding author on reasonable request.
