# Supplementary material for: Quasi-homogenous photocatalysis of quantum-sized Fe-doped TiO2 in optically transparent aqueous dispersions
Source: Sci Rep. 2021 Sep 3;11:17687. doi: 10.1038/s41598-021-96911-6 (PMC8417263; doi:10.1038/s41598-021-96911-6)
Supplement: Supplementary file 1 — Supplementary Figures. [file 41598_2021_96911_MOESM1_ESM.docx]

Supporting Information

Quasi-homogenous photocatalysis of quantum-sized Fe-doped TiO_2_ in optically transparent aqueous dispersions

Marcus Einert,^1,^* Pascal Hartmann,^2^ Bernd Smarsly^2^ & Torsten Brezesinski^3^

^1^Surface Science Laboratory, Department of Materials and Earth Sciences, Technical University of Darmstadt, Otto-Berndt-Str. 3, 64287 Darmstadt, Germany.

^2^Institute of Physical Chemistry, Justus-Liebig-University Giessen, Heinrich-Buff Ring 17, 35392 Giessen, Germany.

^3^Institute of Nanotechnology, Karlsruhe Institute of Technology (KIT), Hermann-von-Helmholtz-Platz 1, 76344, Eggenstein-Leopoldshafen, Germany.

*E-mail: [meinert@surface.tu-darmstadt.de](mailto:meinert@surface.tu-darmstadt.de)


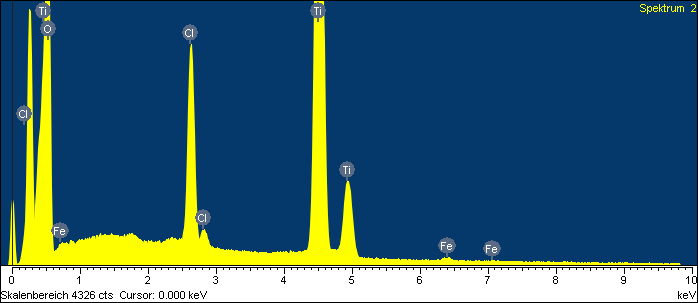


**Figure S1.** EDX spectrum of the (as-prepared) 1.5 mol-% Fe^3+^-doped TiO_2_ nanocrystals.


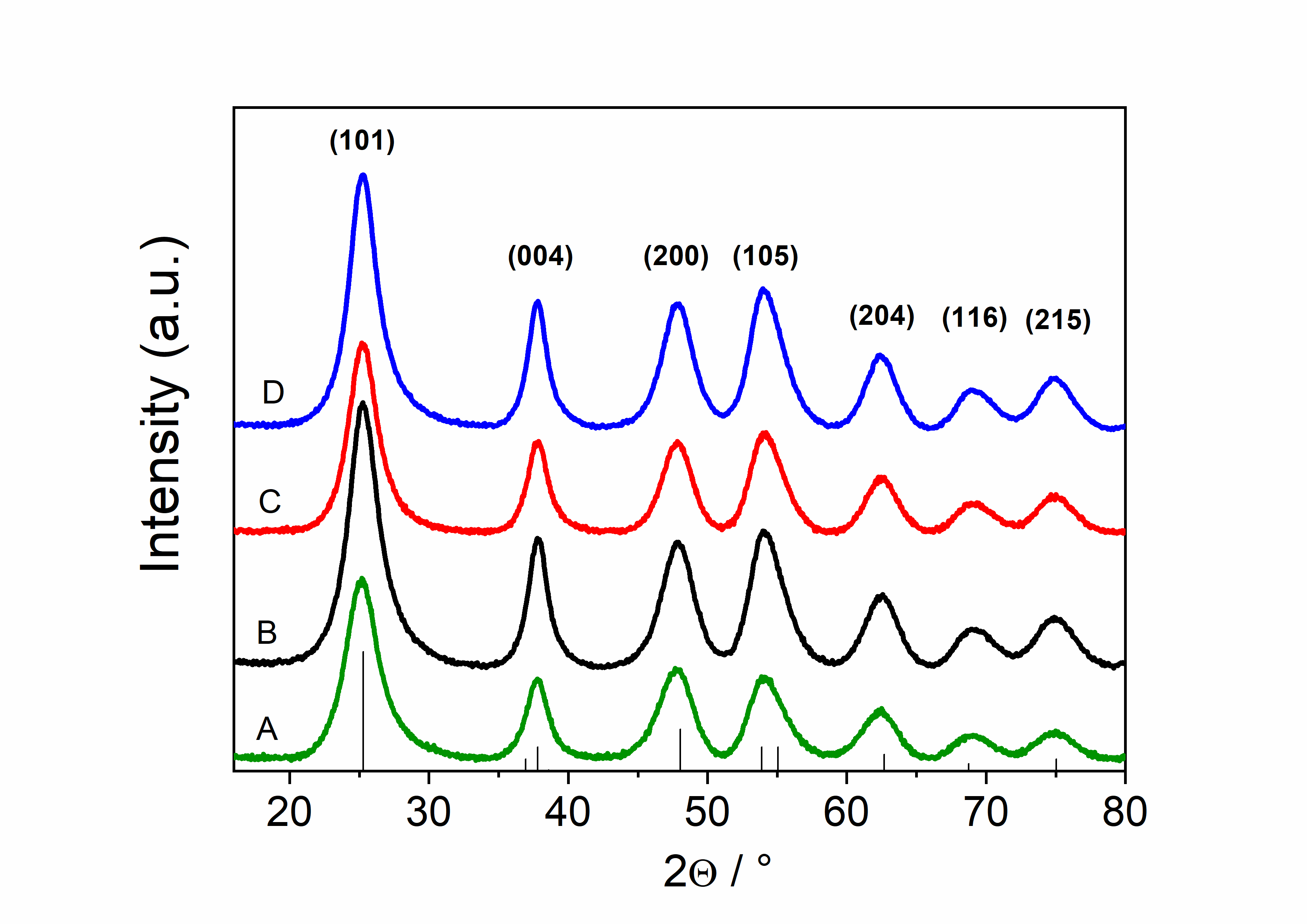


**Figure S2.** XRD patterns of the undoped (A) and 1.0 mol-% (B), 1.5 mol-% (C) and 4.3 mol-% Fe^3+^-doped (D) TiO_2_ nanocrystals.


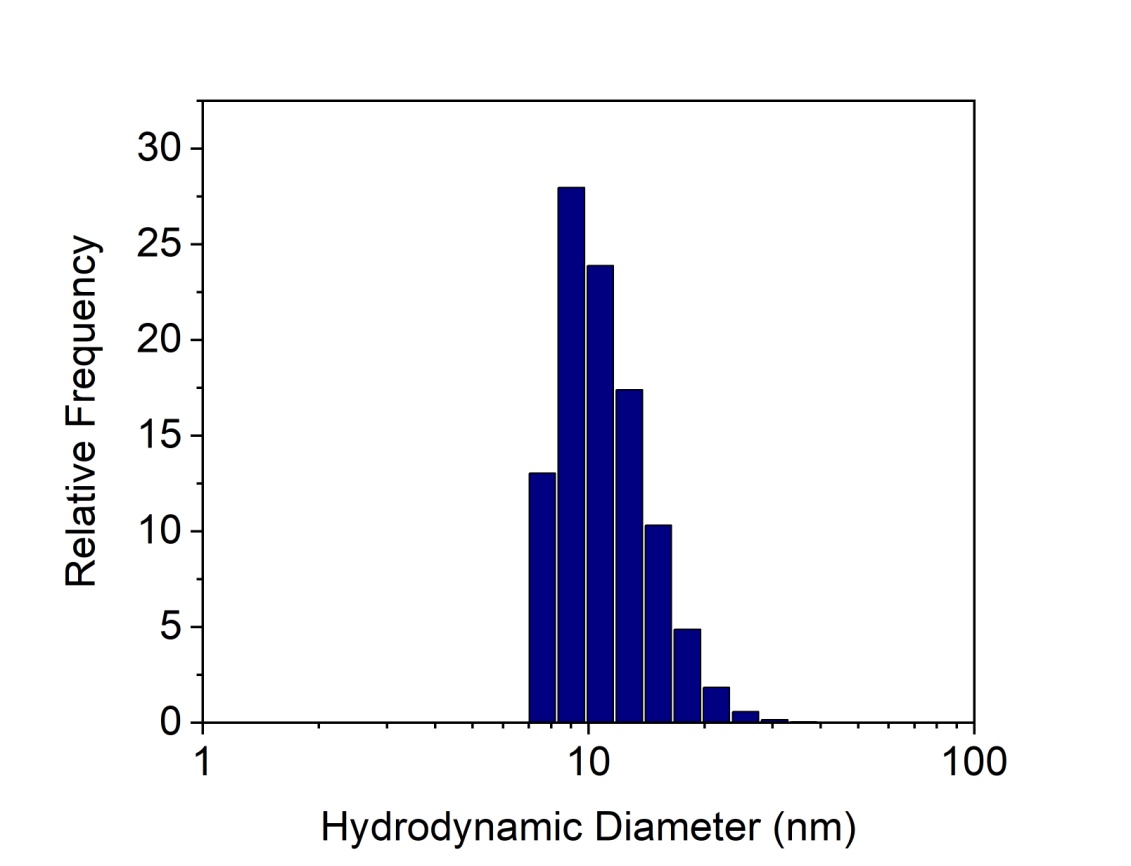


**Figure S3.** Particle-size distribution from DLS for the undoped TiO_2_ nanocrystals dispersed in water.


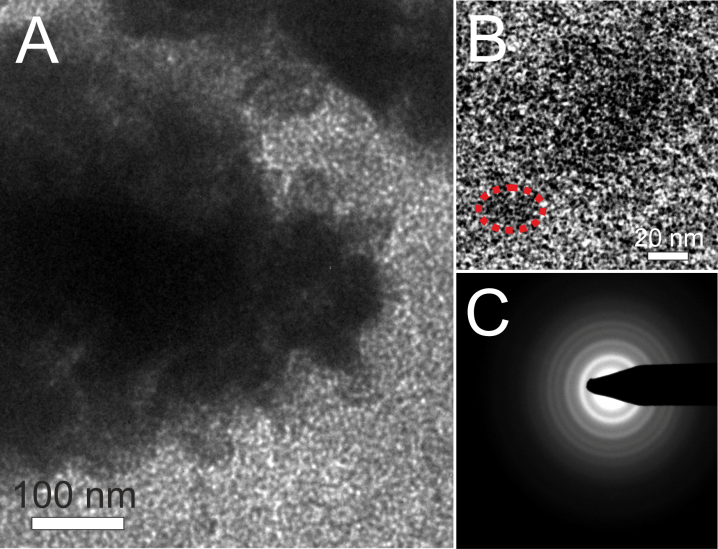


**Figure S4.** (A, B) Bright-field TEM images at different magnifications of the TiO_2_ nanocrystals and (C) corresponding SAED pattern showing rings characteristic of (polycrystalline) anatase TiO_2_.


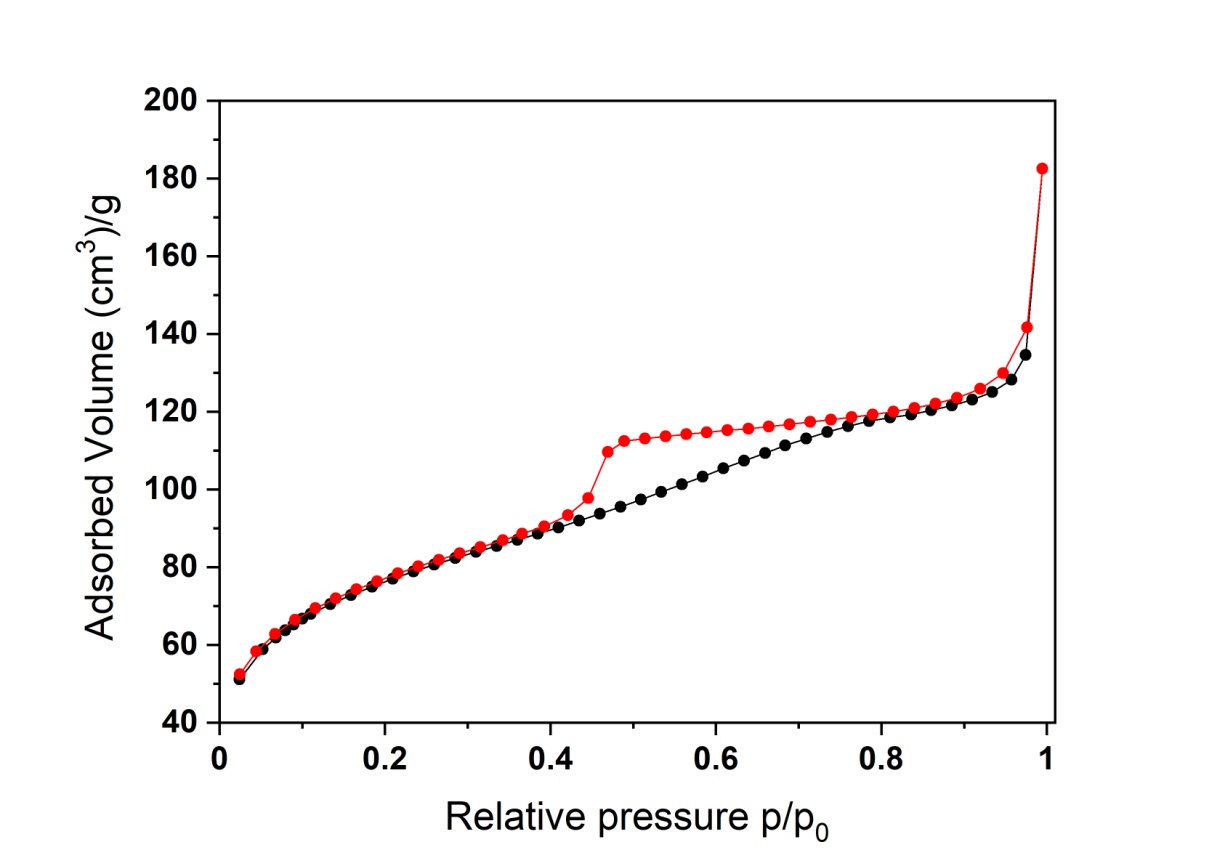


**Figure S5.** N_2_-adsorption/desorption isotherms for the undoped TiO_2_ nanocrystals.


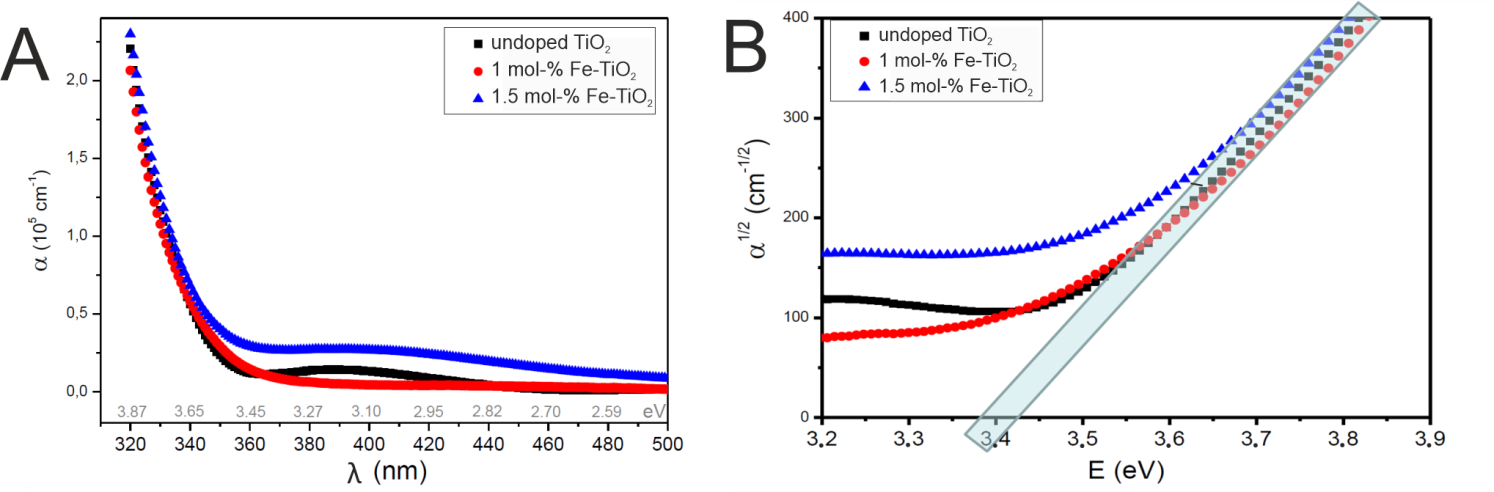


**Figure S6.** (A) Absorbance spectra for the undoped (black) and 1.0 mol-% (red) and 1.5 mol-% (blue) Fe^3+^-doped TiO_2_ nanocrystals and (B) corresponding Tauc plots assuming an indirect optical transition.


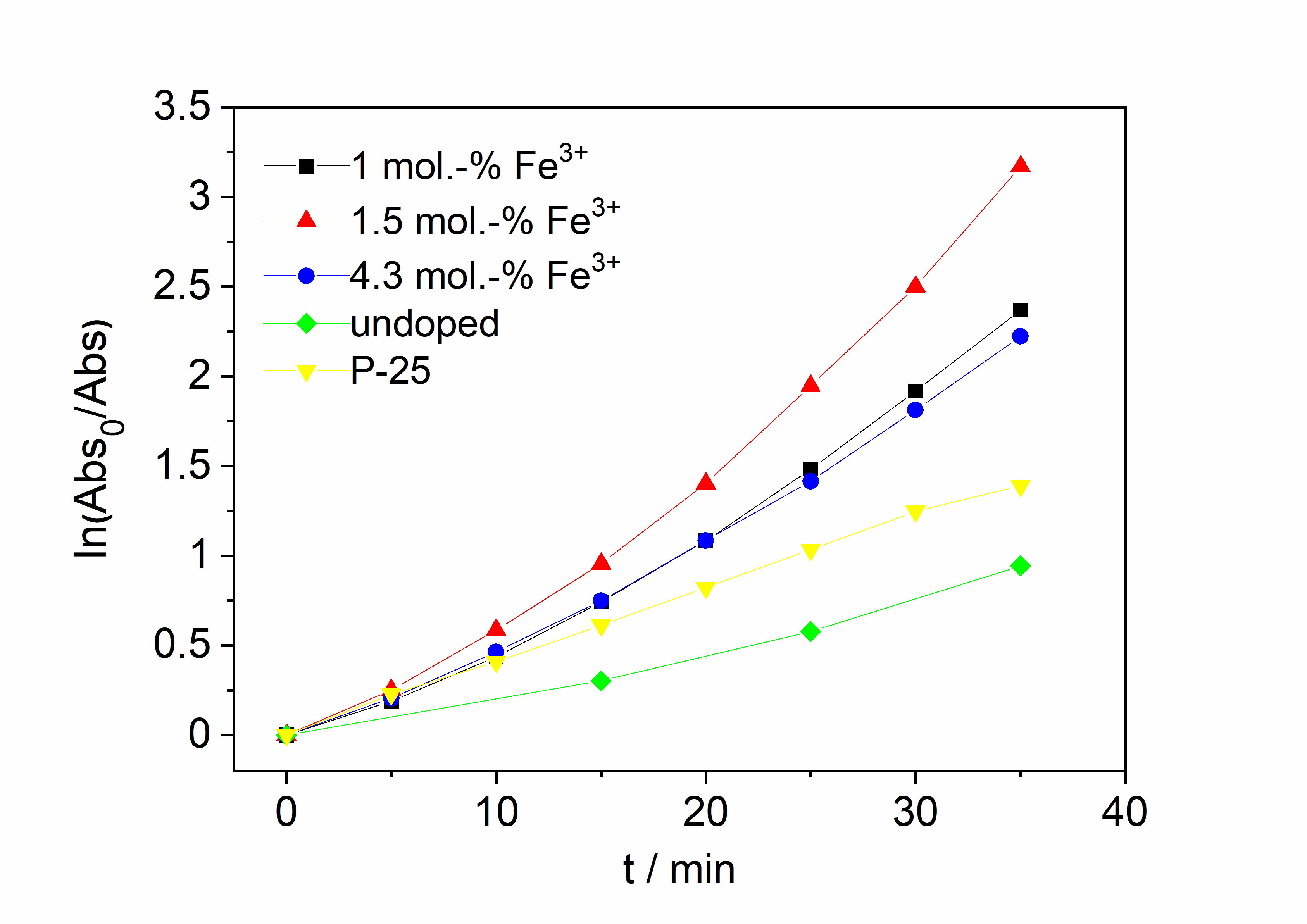


**Figure S7.** Comparison of semilogarithmic plots for the photobleaching of 40 mL aqueous RhB solutions (25 μmol/L) in the presence of P-25 as a reference TiO_2_ photocatalyst (yellow) or undoped (green), 1.0 mol-% (black), 1.5 mol-% (red) and 4.3 mol-% (blue) Fe^3+^-doped TiO_2_ nanocrystals upon UV-light irradiation (35 min in total, 5 min steps). The connecting lines are for eye guidance.


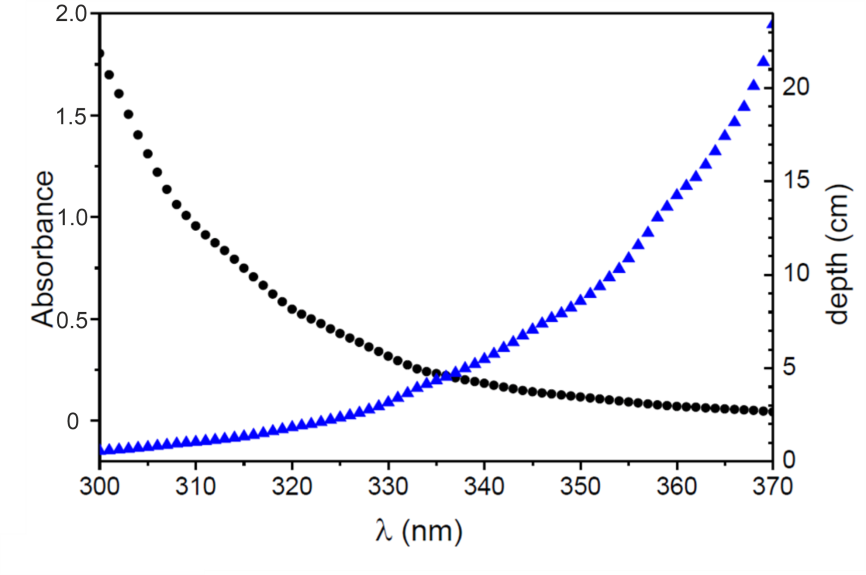


**Figure S8.** Absorbance of incident photons (black) and their penetration depth (blue) in 80 mL aqueous RhB solution (25 μmol/L) as function of the wavelength.


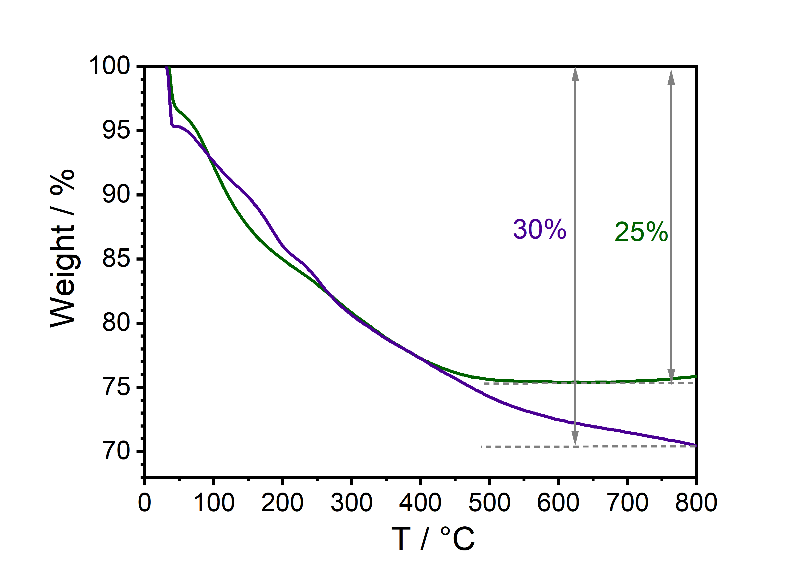


**Figure S9.** TGA curves for the as-prepared (purple) and UV-light irradiated (green) TiO_2_ nanocrystals.


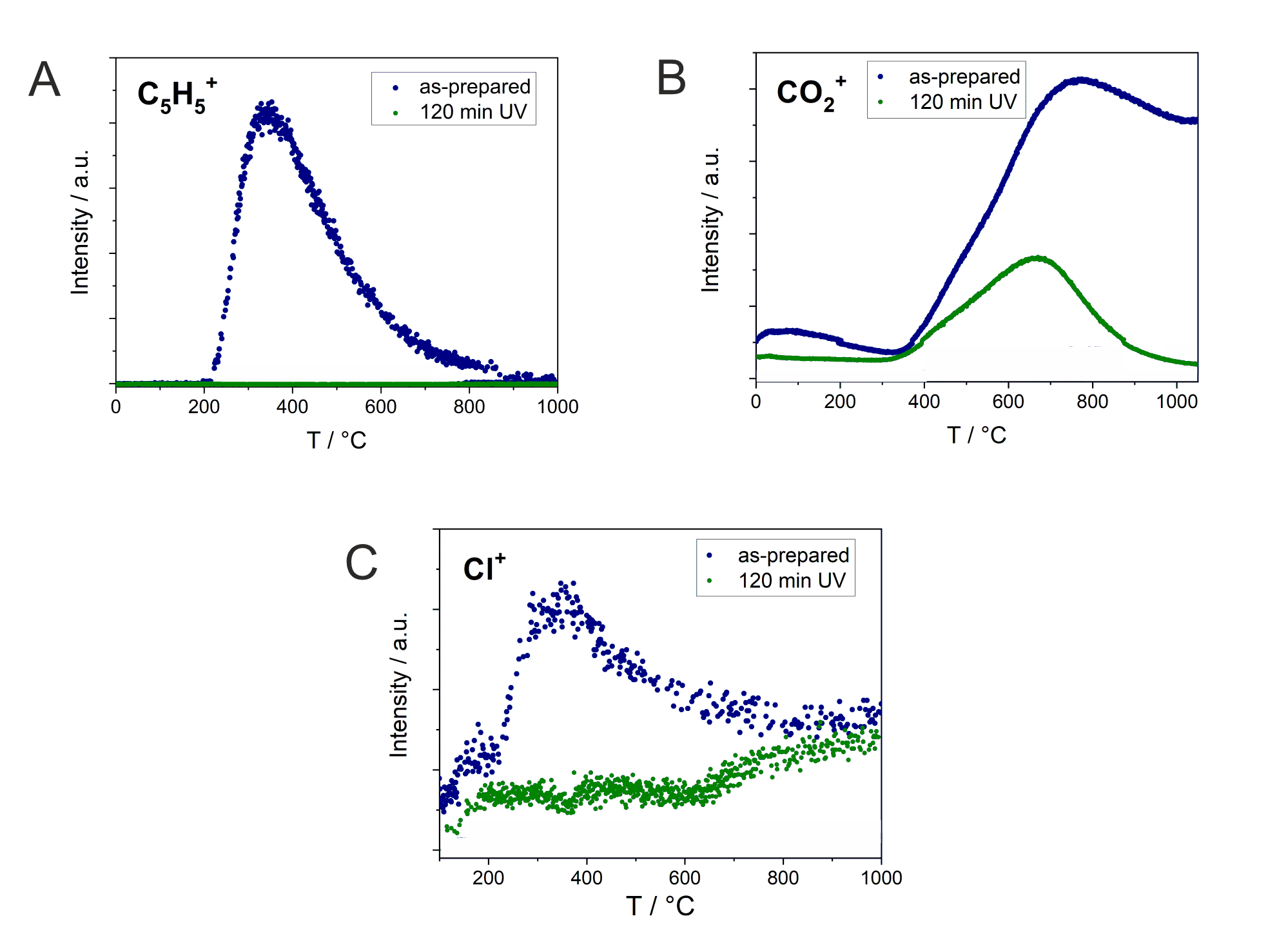


**Figure S10.** MS analysis of the as-prepared (purple) and 120 min UV-treated (green) TiO_2_ nanocrystals upon heating to 1000 °C in an oxidative atmosphere. A) *m*/*z* = 65, B) *m*/*z* = 44 and C) *m*/*z* = 35.
